# The Efficacy of Low-Kilovoltage X-Rays Intraoperative Radiation as Boost for Breast Cancer: A Systematic Review and Meta-Analysis

**DOI:** 10.1155/2023/9035266

**Published:** 2023-07-03

**Authors:** Yuanjian Fan, Ruiwan Chen, Ying Lu, Ying Lin, Yunjian Zhang, Nan Shao, Shenming Wang, Dahong Nie, Zhen Shan

**Affiliations:** ^1^Center of Vascular-Thyroid-Breast Surgery, The First Affiliated Hospital of Sun Yat-Sen University, Guangzhou, China; ^2^Department of Radiology, The First Affiliated Hospital of Sun Yat-Sen University, Guangzhou, China; ^3^Department of Ultrasound, The First Affiliated Hospital of Sun Yat-Sen University, Guangzhou, China

## Abstract

**Background:**

Intraoperative radiotherapy (IORT) is a novel promising technology that may replace external beam radiation therapy (EBRT) as boost for patients receiving breast-conserving surgery. To better evaluate the efficacy of IORT using low-kilovoltage (low-kV) X-rays as boost, we presented this meta-analysis according to the PRISMA checklist.

**Methods:**

Studies reported survival outcomes of intraoperative radiation using low-kilovoltage X-rays system (Intrabeam®, Carl Zeiss Meditec, Dublin, CA, USA) as boost were identified through electronic bibliographic database: PUBMED. The meta-analysis module in Stata (16.0) is used to pool the studies. A Poisson regression model is used to predict a 5-year local recurrence rate.

**Results:**

Twelve studies including 3006 cases were included in the final analysis, with a median follow-up of 55 months weighted by sample size. The pooled local recurrence rate is 0.39% per person-year (95% CI: 0.15%–0.71%), with a low degree of heterogeneity (*I*^2^ = 0%). The predicted 5-year local recurrence rate was 3.45%. No difference in pooled local recurrence rate was found between non-neoadjuvant patients studies and neoadjuvant patients studies (0.41% per person-year vs. 0.58% per person-year, *P* = 0.580).

**Conclusions:**

This study shows that low-kV IORT is an effective method as boost in breast cancer patients, with a low pooled local recurrence rate and low predicted 5-year local recurrence rate. Besides, no difference in the local recurrence rate was found between non-neoadjuvant patients studies and neoadjuvant patients studies. Low-kV IORT boost may be a promising alternative to EBRT boost in the future, which is being tested in the ongoing TARGIT-B trial.

## 1. Introduction

Worldwide, breast cancer has been the most common carcinoma in the women population [1]. The treatments of breast cancer mainly include locoregional treatment and systematic treatment. Breast-conserving surgery (BCS) combined with radiotherapy has been proven to be an effective locoregional treatment and widely accepted since 1985 [2, 3]. In clinical practice, whole breast irradiation with or without boost is the standard radiotherapy treatment after BCS. A phase III randomized trial indicated that the boost group has a lower 20-year cumulative incidence of ipsilateral breast tumor recurrence than the no-boost group (12.0% vs. 16.4%) [4]. Traditionally, external beam radiation therapy (EBRT) was used to deliver boost dose to the tumor bed, taking 5–7 days generally.

Recently, intraoperative radiotherapy (IORT) emerged as an optional method for tumor bed boost which can be performed concurrently with surgery. With an applicator placed in the tumor bed after lumpectomy, IORT can deliver the prescribed dose to the breast tissue. The Intrabeam® system (Carl Zeiss Meditec, Dublin, CA, USA) is one of the IORT equipment that uses low-kilovoltage (low-kV) X-rays. TARGIT-A (NCT00983684) was a large randomized, noninferiority trial that proved low-kV X-ray IORT was an effective alternative to EBRT after BCS, with comparable long-term efficacy for cancer control. At 12-year follow-up, the nonbreast cancer mortality was significantly lower with low-kV IORT (5.41% vs. 9.85%, HR = 0.59, *P* = 0.005), mainly due to fewer deaths from cardiovascular disease, lung problems, and other cancers [5, 6].

The initial series of patients treated with low-kV IORT as an intraoperative boost suggested that it might provide superior local control rates for BCS [7]. The TARGIT-B randomized clinical trial [8], currently recruiting in 38 centers, is comparing low-kV IORT boost with EBRT boost. This trial is testing whether low-kV IORT boost is superior to EBRT boost in terms of local control and survival.

This study aims to pool the low-kV IORT boost studies into a meta-analysis, enriching the population of the sample so as to assess the efficacy of low-kV IORT as boost. We presented this article in accordance with the PRISMA reporting checklist (Supplementary file).

## 2. Methods

### 2.1. Evidence Acquisition

A prospective protocol of objectives, literature-search strategies, inclusion and exclusion criteria, outcome measurements, and methods of statistical analysis was prepared according to the Preferred Reporting Items for Systematic Reviews and Meta-analysis and Meta-analysis of Observational Studies in Epidemiology recommendations for study reporting [9, 10]. Neither the review nor protocol was registered before.

### 2.2. Search Strategy and Selection Criteria

Relevant publications between Jan 1, 2001, and June 1, 2023, were searched through an electronic bibliographic database: PUBMED. The search string for PUBMED contained the following: (((IORT[Title/Abstract]) OR intraoperative radiotherapy [Title/Abstract]) AND breast [Title/Abstract]) AND boost [Title/Abstract]. We also searched relevant reference lists and relevant journals by hand and corresponded with authors. Unpublished studies were also included.

Studies had to meet two criteria for inclusion. They should have investigated recurrence data of breast cancer patients and have used low-kilovoltage X-rays system (Intrabeam®, Carl Zeiss Meditec, Dublin, CA, USA) as intraoperative boost radiation. To sum up, reviews, case reports, and studies use other IORT systems, and studies not using IORT as boost, and studies without recurrence data. Besides, studies not included by Jounral Citation Reports (JCR) were all excluded. To further supplement the database searches, a review of each included study was completed. When multiple reports describing the same population were published, the most recent or complete report was used. Two reviewers (Yuanjian Fan and Zhen Shan) screened each record, and each report was retrieved independently.

### 2.3. Data Extraction

Two reviewers (Yuanjian Fan and Zhen Shan) extracted the data and summarized it independently. Any disagreement was resolved by the adjudicating senior authors. Since the included studies are all nonrandomized, we used the Newcastle-Ottawa Scale (NOS) to assess the quality of all studies, which consists of three parts: selection, comparability, and outcome [11].

In this meta-analysis, the main outcome of interest was the local recurrence rate (LRR). We further investigated the included studies and defined any local recurrence as events. Data were extracted from each of the included studies regarding the characteristics related to the study, protocol, and patients.

### 2.4. Statistical Analysis

We used meta-analysis to provide a pooled summary of the data on the local recurrence rate. To obtain a pooled estimate rate of “events,” we used the Metaprop module in Stata (Version 16.0). The original pooled estimate LRR was counted in per person-year, which means LRR for every single patient in every single year. A randomized-effect meta-analysis of proportion models was used to estimate an overall LRR. Of note, we specifically calculate the pooled LRR within two subgroups: non-NAT (neoadjuvant treatment) patients studies and NAT patients studies, to further assess the efficacy of IORT as boost in different subgroups.

Because the majority of the studies did not follow up for over 5 years, it was difficult to estimate a reliable 5-year LRR. Therefore, Poisson regression modeling for the pooled recurrence rate was used to estimate a reliable 5-year LRR [12].

### 2.5. Heterogeneity

We estimated the heterogeneity between studies with the *I*^2^ statistic, which described the percentage of variation between studies due to heterogeneity rather than chance. The values of 25%, 50%, and 75% show low, moderate, and high degrees of heterogeneity, respectively [13]. To draw a conclusion that can be extrapolated to more breast cancer patients, we used a random-effects meta-analysis proportions model to calculate the LRR.

## 3. Results

### 3.1. Searching, Inclusion, and Exclusion

**Figure 1:**
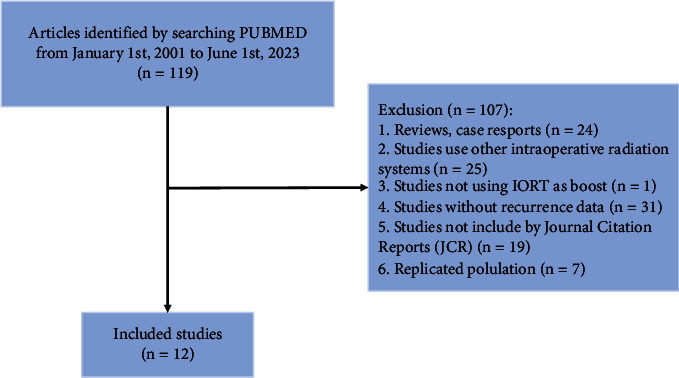
Flow diagram of studies identifying inclusion and exclusion.

The PubMed search string generated 119 results. After examining the inclusion and exclusion criteria, eleven retrospective studies (11 cohort studies and 1 case-control study) were considered eligible to be included [7, 14–24] (Figure 1). No risk of bias was found due to missing results or each synthesis.

### 3.2. Included Studies and Patients' Characteristics

Eventually, 12 studies including 3006 cases were included in the final analysis, with a median follow-up of 55 months weighted by sample size (Table 1 and Figure 1). Patients in 12 studies were aged from over 18 to 86. The median follow-up duration of 12 studies ranged from 23.3 months to 91.5 months. The tumor size stages were T1-T2 in 6 of included studies, while 4 of the studies included T2+ tumors and 1 of the studies included pCR and Tis tumor. The lymph node statuses were also available, ranging from N0 to N3 in 8 studies, N0-1 in 2 of the studies, and only N0 patients in 1 of the studies. Tumor grades were available in 8 of 12 studies, ranging from grade 1 to grade 3. Only 1 of the studies (Stoian et al.) was with unavailable characteristics such as age, tumor size, lymph node status, and tumor grade. Besides, most patients underwent breast-conserving surgery and received similar system treatments, including endocrine therapy (if HR-positive) and chemotherapy. The majority of the studies (7 of 12) only include non-NAT patients, while 1 study (Kolberg et al.) only includes NAT patients. Four of the studies include a very small proportion of NAT patients (13.9% in Cho et al., 4.2% in Stoian et al., 11.18% in Sarria et al., and 14.7% in Hochhertz et al.), and 3 of them have not reported recurrence data of NAT patients specifically, therefore regarded as non-NAT patient study in our analysis. Only 1 study (Cho et al.) reported the recurrence data of NAT patients with no local recurrence in 91 (13.9%) NAT patients. Therefore, the LRR of IORT as boost in non-NAT patients and NAT patients in this study is pooled in different subgroups, respectively. No missing results were stated in the studies. Quality of all studies is assessed by the Newcastle-Ottawa Scale (NOS), as shown in Tables 2 and 3.

### 3.3. Local Recurrence Rate

**Figure 2:**
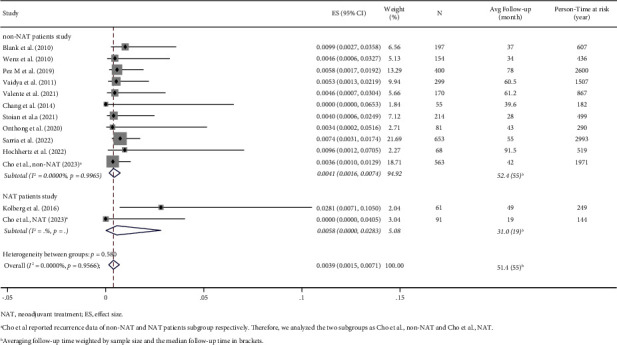
Forrest plot of the pooled local recurrence rate of 12 studies.

**Figure 3:**
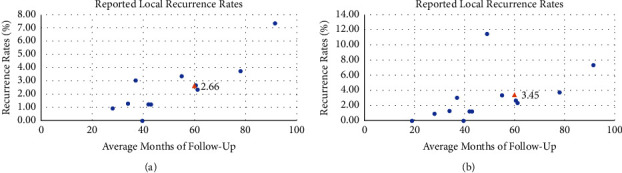
Predicted the 5-year local recurrence rate using the Poisson regression model. Each blue circle represents one study. (a) The estimated 5-year recurrence rate (orange triangle) with 11 non-NAT literature studies using the Poisson regression model is 2.66% (95% CI: 0% to 12.80%). (b) The estimated 5-year recurrence rate (orange triangle) with all 13 literature studies using the Poisson regression model is 3.45% (95% CI: 0% to 14.30%).

Overall, the pooled LRR of 12 studies is 0.39% per person-year (95% confidence interval (CI): 0.15%–0.71%). The overall *I*^2^ index was low (*I*^2^ = 0.00%) in considering the characteristics of subpopulations investigated and study designs. The predicted 5-year LRR of low-kV IORT boost estimated by the Poisson regression model is 3.45% (95% CI: 0%–14.30%) (Figures 2 and 3(a)).

For non-NAT patients, the pooled LRR of low-kV IORT-IORT boost is 0.41% per person-year (95% CI: 0.16%–0.74%) for the 11 non-NAT patients studies, with a low heterogeneity (Figure 2). The predicted 5-year LRR of low-kV IORT boost in non-NAT patients is 2.66% (95% CI: 0%–12.80%) (Figure 3(b)).

Only two studies report the recurrence data of low-kV IORT as boost in NAT patients, with a pooled LRR of 0.58% per person-year (95% CI: 0%–2.83%) (Figure 2). No difference in the pooled local recurrence rate was found between non-NAT patients studies and NAT patients studies (0.41% per person-year vs. 0.58% per person-year, *P* = 0.580).

## 4. Discussion

The advantages of using low-kV IORT include the ability to visualize the tumor bed directly. Surgeons can deliver a single dose of radiation to the surrounding tissue intraoperatively, ensuring the treatment of the high-risk tissue and eliminating the risk of marginal missing. Patients who undergo IORT boost can omit postoperative EBRT boost which may cost 5–7 days generally. Besides, the cosmetic and toxicity outcomes of low-kV IORT boost are good because of the lower doses [15, 25]. In the TARIGIT-A trial, there was no significant difference in any protocol-defined wound-related complications such as fibrosis, breast edema, retraction, ulceration, lymphedema, hyperpigmentation, and pain. Fewer grade 3 or 4 radiotherapy-related skin complications are associated with low-kV IORT patients than with EBRT (4/1721 vs. 13/1730, *P* = 0.029) [5].

Both the pooled LRR (0.41%) and the predicted 5-year LRR (3.45%) are relatively low in overall patients. When NAT patients' studies were excluded, we may achieve a relatively lower predicted 5-year LRR than overall studies (2.66% vs. 3.45%). The previous study reported the 5-year LRR of 4.3% (95% CI: 3.8%–4.7%) in patients who received BCS plus EBRT boost [26], which seems to be higher than that of IORT as boost in our study. However, such differences may refer to the improvement of adjuvant therapy.

A meta-analysis carried by Early Breast Cancer Trialists' Collaborative Group (EBCTCG) indicates that tumors downsized by NAT might be associated with a higher local recurrence risk after BCS, comparing to tumors of the same dimensions in patients who received adjuvant chemotherapy instead [27]. Multiple reasons may contribute to the higher local recurrence rate for NAT patients. Firstly, NAT is usually prescribed to those patients with high risks, such as large tumor size, high tumor grade, lymph node involved, HER2 positive, or triple-negative disease. These features make the prognosis worse than that of non-NAT patients. Secondly, it is difficult to localize the primary tumor bed precisely after NAT, especially for tumor with good response. This may lead to the high risk of missing tumor bed irradiation. In our study, the local recurrence rate of non-NAT patients studies and NAT patients studies showed no significant difference (0.41% per person-year vs. 0.58% per person-year, *P*=0.580). However, it is a far cry from the LRR of two included non-NAT patients' studies (2.81% per person-year vs. 0% per person-year). We supposed that such difference mainly refers to the patients' characteristics. Although the exact NAT patients' characteristics are unavailable in Cho et al., more than half of the patients (33 of 61) in Kolberg et al. suffered from positive lymph node. Besides, high proportion of HER2 positive patients may also be relevant to high LRR in Kolberg et al. The result of Kolberg et al. showed that low-kV IORT boost is superior to EBRT boost in NAT patients (local recurrence-free survival 88.5% vs. 79.9%). The efficacy of low-kV IORT boost in NAT patients needs more validation, as is being done in the TARGIT-B randomized trial (NCT01792726) [8].

Intraoperative electron radiotherapy (IOERT) as boost has been proved to be an efficacy radiotherapy prior to WBI, with outstanding local control rates. In a long-term result of a phase III randomized study included 133 patients using IOERT as boost, only 0.8% of 5-year in-breast true recurrences was observed [28]. A large pooled analysis compared 1109 unselected patients from 7 different centers using the same IOERT and WBI doses: 10 Gy as a boost and 50–54 Gy WBI. At a median follow-up of 72.4 months, 99.2% of the tumor control rate was achieved [29]. In our study, only 4 of the included studies reach a median follow-up time for over 5 years: Pez M et al., Vaidya et al., Valente et al., and Hochhertz et al. The LRR of them are 3.75% (15/400), 2.68% (8/299), 2.35% (4/170), and 7.35% (5/68), respectively. All of them are higher than that of IOERT as boost. Head-to-head studies comparing low-kV IORT boost, EBRT boost, and IOERT boost are necessary to further compare the efficacy of multiple methods of boost.

## 5. Limitations

There are several limitations in our study. Firstly, all the included studies are nonrandomized studies, leading to unavoidable selection bias. Besides, all studies were carried out in different clinical centers, which may result in different IORT protocols. Thirdly, most of these studies (10 of 12) are single-armed studies and have no control group, which make it difficult to compare low-kV IORT with other boost methods.

## 6. Conclusion

This study shows that low-kV IORT is an effective method as boost in breast cancer patients, with a low pooled local recurrence rate and a low predicted 5-year local recurrence rate. Besides, no difference of the local recurrence rate was found between non-neoadjuvant patients' studies and neoadjuvant patients' studies. Low-kV IORT boost may be a promising alternative to EBRT boost in the future, which is being tested in the ongoing TARGIT-B trial.

## Figures and Tables

**Table 1:** Studies and patients' characteristics.

Author	Year	Study type	NAT or non-NATpatient study	No. of patients	No. of events	Age (years)	Median follow-up(months)	Tumor size	Lymph node status	Tumor grade	Boost dose
Blank et al.	2010	Cohort	Non-NAT	197	6	30–84	37	T1-2	N0–3	G1–3	20 Gy
Wenz et al.	2010	Cohort	Non-NAT	154	2	30–83	34	T1-2	N0–3	NA	20 Gy
Kolberg et al.	2016	Cohort	NAT	61	7	<45–≥65	49	T1-2	N0–3	G1–3	20 Gy
Pez M et al.	2019	Cohort	Non-NAT	400	15	30–85	78	T1-2	N0–3	G1–3	20 Gy
Vaidya et al.	2011	Case-control	Non-NAT	299	8	28–83	60.5	T1-2	N0–3	G1–3	20 Gy
Valente et al.	2021	Cohort	Non-NAT	170	4	38–87	61.2	T1–3+	N0–3	G1–3	20 Gy
Chang et al.	2014	Cohort	Non-NAT	55	0	39–83	39.6	T1-2	N0	NA	5 Gy
Stoian et al.^a^	2021	Cohort	Both	214	2	NA	28	NA	NA	NA	20 Gy
Onthong et al.	2020	Cohort	Non-NAT	81	1	30–>70	43	T1–3	N0-N1+	G1–3	20 Gy
Sarria et al.^a^	2022	Cohort	Both	653	22	>18	55	T1–3	N0-1	G1–3	6−20 Gy
Hochhertz et al.^a^	2022	Cohort	Both	68	5	37.8–79.3	91.5	T1–4	N0–3	NA	20 Gy
Cho et al.^b^	2023	Cohort	Both	654	7	27–87	42	pCR-T2	N0–3	G1–3	20 Gy

NAT, neoadjuvant treatment; non-NAT, non-neoadjuvant treatment; NA, not available. ^a^These studies include a small proportion of NAT patients (4.2% in Stoian et al., 11.18% in Sarria et al., and 14.7% in Hochhetz et al.) but have not reported the recurrence data of NAT patients specifically. Therefore, we regarded these studies as non-NAT patient studies in our analysis. ^b^Cho et al. reported recurrence data of non-NAT and NAT patient subgroups, respectively. Therefore, we analyzed the two subgroups as Cho et al., non-NAT, and Cho et al., NAT.

**Table 2:** Assessment of the case-control study with the Newcastle-Ottawa Scale (NOS).

Study	Selection				Comparability^*∗*^	Exposure			Quality score
	Adequate definition	Representative cases	Selection of controls	Definition of controls		Ascertainment of exposure	Same method of ascertainment	Nonresponse rate	
Vaidya et al.	★	★	★	★	★☆	★	★	★	★★★★★★★★

^*∗*^Comparability variables: 1 = age; 2 = tumor size; 3 = lymph node status; 4 = tumor grade; 5 = hormone receptor status; 6 = HER2 status. If all characteristics were comparable, two stars; if two or three characteristics were comparable, one star; otherwise, no star.

**Table 3:** Assessment of cohort studies with the Newcastle-Ottawa Scale (NOS).

Study	Selection				Comparability^*∗*^	Outcome			Quality score
	Representative treatment group	Representative reference group	Assignment for treatment	Outcome unpresented		Assessment of outcome	Long enough follow-up	Adequate follow-up	
Blank et al.	★	☆	★	★	☆☆	★	☆	★	★★★★★
Wenz et al.	★	☆	★	★	☆☆	★	☆	★	★★★★★
Kolburg et al.	☆	★	★	★	★★	★	☆	★	★★★★★★★
Pez M et al.	★	☆	★	★	☆☆	★	★	★	★★★★★★
Valente et al.	★	★	★	★	★★	★	★	★	★★★★★★★★★
Chang et al.	★	☆	★	★	☆☆	★	☆	★	★★★★★
Stoian et al.	★	☆	★	★	☆☆	★	☆	★	★★★★★
Onthong et al.	★	☆	★	★	☆☆	★	☆	★	★★★★★
Sarria et al.	★	☆	★	★	☆☆	★	☆	★	★★★★★
Hochhertz et al.	★	☆	★	★	☆☆	★	★	★	★★★★★★
Cho et al.	★	☆	★	★	☆☆	★	☆	★	★★★★★

^*∗*^Comparability variables: 1 = age; 2 = tumor size; 3 = lymph node status; 4 = tumor grade; 5 = hormone receptor status; 6 = HER2 status. If all characteristics were comparable, two stars; if two or three characteristics were comparable, one star; otherwise, no star.

## Data Availability

All the data in our study can be accessed from the 12 included studies of meta-analysis.

## Additional Points

Reporting checklist. The authors have completed the PRSIMA reporting checklist (Supplementary file).

## Supplementary Materials

Supplementary Materials Supplementary file. The PRISMA 2020 reporting checklist: a guideline for reporting systematic reviews.
